# Supplementary material for: The COVID University Challenge: A Hazard Analysis of Critical Control Points Assessment of the Return of Students to Higher Education Establishments
Source: Risk Anal. 2021 Jun 2;41(12):2286–92. doi: 10.1111/risa.13741 (PMC8242865; doi:10.1111/risa.13741)
Supplement: Supplementary file 1 — Table I. Summary of the high‐risk activities identified through a Hazard Analysis of Critical Control Point (HACCP) assessment for the return of students to an on‐campus university setting during the COVID‐19 pandemic. [file RISA-41-2286-s001.docx]

Table I. Summary of the high risk activities identified through a Hazard Analysis of Critical Control Point (HACCP) assessment for the return of students to an on-campus university setting during the COVID-19 pandemic. This HACCP assessment considers the transmission of SARS-CoV-2 between individuals as the hazard under investigation.

| **Critical Control Point** | **Level of concern (high, medium, low)** | **Proposed Critical Limits and Recommendations** |
| --- | --- | --- |
| **1. Preventing arrival of virus in to the student household** | High | Ensure well-ventilated environment when interacting with individuals from outside the student household, outdoor interactions are lower risk than indoors (Qian et al., 2020; WHO, 2020a).  Wash hands with soap and water for at least 20 seconds after returning to student household. |
| **2. Shared car/minibus journey (including shared transport to placements, sporting events, field courses, social events etc.)** | High | Sit as far from others as possible, particularly avoiding sitting directly face to face with another passenger (UK Government, 2020a).  Open windows to increase ventilation (CDC, 2020a; GHHIN, 2020; Morawska & Milton, 2020; Qian et al., 2020; UK Government, 2020b, WHO, 2020a).  Wear face coverings in accordance with current national and university policy and guidance (WHO, 2020b).  Minimise journey time and where possible, keep journeys under 15 minutes in duration (ECDC, 2020; UK Government, 2020b). |
| **3. Returning to university household after time (overnight) away** | High | Self-isolate if had contact with confirmed case or told to self-isolate by public health officials or those working for their Test and Trace programmes (UK Government, 2020c).  Follow local guidance for the wearing of face coverings (WHO, 2020b).  Ensure good hand hygiene and washing practices (CDC, 2020b).  Maintain a minimum social distance of 2m as much as possible or at least 1m if other mitigations measures are in place, such as wearing face coverings (UK Government, 2020d).  Ensure well-ventilated environment (CDC, 2020a; GHHIN, 2020; Morawska & Milton, 2020; Qian et al., 2020; UK Government, 2020b, WHO, 2020a).  Ensure clear guidance is available for students to follow. |
| **4. Interaction with people beyond university household (this includes the students having different placement groups to their other household members)** | High | Adhere to rules about group sizes (e.g. “Rule of six” implemented in September 2020) when socializing. Where possible minimize the number of university households involved in the interactions (Nielsen & Sneppen, 2020).  Minimise contact time between individuals and where possible keep interactions shorter than 15 minutes in duration (ECDC, 2020).  Ensure well-ventilated environment with windows open fully as much as possible (CDC, 2020a; GHHIN, 2020; Morawska & Milton, 2020; Qian et al., 2020; UK Government, 2020b, WHO, 2020a).  Maintain a minimum social distance of 2m as much as possible or at least 1m if other mitigations measures are in place, such as wearing face coverings (UK Government, 2020d).  Wash hands with soap and water for at least 20 seconds after returning to student household. |
| **5. Sharing of personal items** | High – Medium depending on what items are being shared | Don’t share personal items! If unavoidable try to ensure a minimum of 72 hours between use by individuals from different households (van Doremalen, et al., 2020).  Sanitise items between uses by different individuals (CDC, 2020d; van Doremalen, et al., 2020).  Wash hands with soap and water for at least 20 seconds before and after exchange of items (CDC, 2020b; van Doremalen, et al., 2020). |
| **6. Indoor queueing /crowding** | High | Minimise queueing time by avoiding peak times. Keep queuing time to less than 15 minutes wherever possible (ECDC, 2020).  Observe and use space markers to help judge 2m distance (Nielsen & Sneppen, 2020; UK Government, 2020d; 2m is roughly 2 full adult arm lengths apart (Wikipedia, 2020).  Ensure well-ventilated environment (CDC, 2020a; GHHIN, 2020; Morawska & Milton, 2020; Qian et al., 2020; UK Government, 2020b, WHO, 2020a).  Maintain a minimum social distance of 2m as much as possible or at least 1m if other mitigations measures are in place, such as wearing face coverings (UK Government, 2020d).  Wear face coverings in accordance with current national and university policy and guidance (WHO, 2020b).  Wash hands with soap and water for at least 20 seconds before and after queuing (CDC, 2020b; van Doremalen, et al., 2020). |
| **7. Sharing of study facilities** | High | Disinfect items between uses by different individuals (CDC, 2020c; van Doremalen, et al., 2020).  Ensure well-ventilated environment, open windows as much as possible (CDC, 2020a; GHHIN, 2020; Morawska & Milton, 2020; Qian et al., 2020; UK Government, 2020b, WHO, 2020a).  Maintain a minimum social distance of 2m or at least 1m if other mitigation measures are in place, such as wearing face coverings (UK Government, 2020d).  Wear masks / face coverings in accordance with current national and university policy and guidance (WHO, 2020d) and avoid sitting face-to-face with another individual (UK Government, 2020a).  Wash hands with soap and water for at least 20 seconds before and after using study facilities (CDC, 2020b; van Doremalen, et al., 2020) and avoid touching your face (Macias et al., 2010). |
| **8. Outdoor queuing** | High – low depending on length of queue and time spent queuing | Minimise queueing time by avoiding peak times. Keep queuing time to less than 15 minutes when possible (ECDC, 2020).  Observe and use space markers to help you judge 2m distance coverings (UK Government, 2020d); 2m is roughly 2 full adult arm lengths apart (Wikipedia, 2020).  Maintain a minimum social distance of 2m as much as possible or at least 1m if other mitigations measures are in place, such as wearing face coverings (UK Government, 2020d; WHO, 2020b).  Wash hands with soap and water for at least 20 seconds before and after queuing (CDC, 2020b; van Doremalen, et al., 2020). |
| **9. Sharing of prayer facilities** | High | Adhere to rules about group sizes. Where possible minimize the number of university households involved in the interactions (Nielsen & Sneppen, 2020).  Minimise contact time between individuals, keeping the use of shared spaces to less than 15 minutes when possible (ECDC, 2020) and avoid singing or chanting and where deemed essential, limit the duration of singing (UK Government, 2020e).  Individuals to cleanse prior to attending prayer facility (UK Government, 2020f). If this is not possible, only one individual to use the cleansing room at a time with the room to be thoroughly cleaned and sanitized between uses (UK Government, 2020e; van Doremalen, et al., 2020).  Any items, such as a prayer mat, that are brought in to the facility must also be taken home immediately after use (UK Government, 2020e).  Ensure well-ventilated environment, with windows open fully where possible (CDC, 2020a; GHHIN, 2020; Morawska & Milton, 2020; Qian et al., 2020; UK Government, 2020b, WHO, 2020a).  Maintain a minimum social distance of 2m as much as possible or at least 1m if other mitigations measures are in place, such as wearing face coverings (UK Government, 2020d).  Wear face coverings in accordance with current national and university policy and guidance (WHO, 2020b).  Wash hands with soap and water for at least 20 seconds before and after using prayer facilities (CDC, 2020b; van Doremalen, et al., 2020). |
| **10. Sharing of changing facilities** | High | Adhere to rules about group sizes. Where possible minimize the number of university households involved in the interactions (Nielsen & Sneppen, 2020).  Minimise contact time between individuals, keeping the use of shared spaces to less than 15 minutes when possible (ECDC, 2020).  Do not share personal items. If unavoidable, sanitise shared items before using them (CDC, 2020d; van Doremalen, et al., 2020).  Ensure well-ventilated environment, with windows open fully when and where possible (CDC, 2020a; GHHIN, 2020; Morawska & Milton, 2020; Qian et al., 2020; UK Government, 2020b, WHO, 2020a).  Maintain a minimum social distance of 2m as much as possible or at least 1m if other mitigations measures are in place, such as wearing face coverings (UK Government, 2020d).  Wear face coverings in accordance with current national and university policy and guidance (WHO, 2020b).  Wash hands with soap and water for at least 20 seconds before and after using shared facilities (CDC, 2020b; van Doremalen, et al., 2020). |
| **11. Sharing training/practice facilities** | High | Adhere to rules about group sizes. Where possible minimize the number of university households involved in the interactions (Nielsen & Sneppen, 2020).  Minimise contact time between individuals, keeping the use of shared spaces to less than 15 minutes when possible (ECDC, 2020) and avoid being face-to-face (UK Government, 2020a).  Do not share personal items. If sharing is unavoidable, disinfect items before using them (CDC, 2020d; van Doremalen, et al., 2020).  Ensure well-ventilated environment, with windows open fully where possible (CDC, 2020a; GHHIN, 2020; Morawska & Milton, 2020; Qian et al., 2020; UK Government, 2020b, WHO, 2020a).  Maintain a minimum social distance of 2m as much as possible or at least 1m if other mitigations measures are in place, such as wearing face coverings (UK Government, 2020d).  Wash hands with soap and water for at least 20 seconds before and after using shared facilities (CDC, 2020b; van Doremalen, et al., 2020). |
| **12. Singing/Cheering** | High | Ensure well-ventilated environment, with windows open fully where possible (CDC, 2020a; GHHIN, 2020; Morawska & Milton, 2020; Qian et al., 2020; UK Government, 2020b, WHO, 2020a).  Maintain a minimum social distance of 2m as much as possible or at least 1m if other mitigations measures are in place, such as wearing face coverings (UK Government, 2020d) and stand side-by-side rather than face-to-face (UK Government, 2020a).  Wear face coverings in accordance with current national and university policy and guidance (WHO, 2020b). |
| **13. Participation in conferences, meetings, etc.** | High | Where possible, make use of virtual meeting platforms.  Adhere to rules about group sizes when attending. Where possible minimize the number of university households involved in the interactions (Nielsen & Sneppen, 2020). .  Minimise contact time between individuals, keeping the use of shared spaces to less than 15 minutes when possible (ECDC, 2020).  Ensure well-ventilated environment, with widows open fully where possible (CDC, 2020a; GHHIN, 2020; Morawska & Milton, 2020; Qian et al., 2020; UK Government, 2020b, WHO, 2020a).  Maintain a minimum social distance of 2m as much as possible or at least 1m if other mitigations measures are in place, such as wearing face coverings (UK Government, 2020d).  Wear face coverings in accordance with current national and university policy and guidance (WHO, 2020b).  Wash hands with soap and water for at least 20 seconds regularly throughout conference and before and after in-person meetings (CDC, 2020b; van Doremalen, et al., 2020). |
| **14. Sharing accommodation on field courses** | High | Avoid shared accommodation wherever possible. If unavoidable, adhere to rules about group sizes and where possible, minimize the number of university households involved in the interactions (Nielsen & Sneppen, 2020).  Do not share personal items. If sharing is unavoidable, sanitise any items before using them (CDC, 2020d; van Doremalen, et al., 2020).  Ensure well-ventilated environment, with windows open fully where possible (CDC, 2020a; GHHIN, 2020; Morawska & Milton, 2020; Qian et al., 2020; UK Government, 2020b, WHO, 2020a).  Maintain a minimum social distance of 2m as much as possible or at least 1m if other mitigations measures are in place, such as wearing face coverings (UK Government, 2020d).  Wear face coverings in accordance with current national and university policy and guidance (WHO, 2020b).  Wash hands with soap and water for at least 20 seconds before and after using shared facilities (CDC, 2020b; van Doremalen, et al., 2020).  Students must not attend field course if they have COVID symptoms  If student develops COVID symptoms during field course, isolation rules apply; consult current university and national policies |
| **15. Examining patients** | High-low depending on use of PPE | Examiner to wash hands before and after examining patient (CDC, 2020b; van Doremalen, et al., 2020).  Both examiner and patient to wear a mask and visor where possible (WHO, 2020b) and avoid being face-to-face (UK Government, 2020a).  Wear full PPE when examining the mouth, throat, and nose or when a patient has COVID symptoms (it is highly unlikely students will be allowed to examine such patients or do an Ear Nose Throat exam). |
| **16. Remaining within one room whilst on placement** | High | Ensure well-ventilated environment, with windows open fully where possible (CDC, 2020a; GHHIN, 2020; Morawska & Milton, 2020; Qian et al., 2020; UK Government, 2020b, WHO, 2020a).  Maintain a minimum social distance of 2m as much as possible or at least 1m if other mitigations measures are in place, such as wearing face coverings (UK Government, 2020d) and avoid being face-to-face (UK Government, 2020ab).  Wear face coverings in accordance with current national and university policy and guidance (WHO, 2020b).  Students must not attend placement if they have COVID symptoms  Wash hands with soap and water for at least 20 seconds before and after using shared room (CDC, 2020b; van Doremalen, et al., 2020). |
| **17. Moving around within buildings** | High | Ensure good movement of people throughout shared spaces to minimize likelihood of crowding (Nielsen & Sneppen, 2020).  Minimise queueing/waiting time.  Follow any designated one-way systems and observe and use space markers to help judge 2m distance (UK Government, 2020d); 2m is roughly 2 full adult arm lengths apart (Wikipedia, 2020).  Ensure well-ventilated environment, with windows open fully where possible (CDC, 2020a; GHHIN, 2020; Morawska & Milton, 2020; Qian et al., 2020; UK Government, 2020b, WHO, 2020a).  Maintain a minimum social distance of 2m as much as possible or at least 1m if other mitigations measures are in place, such as wearing face coverings (UK Government, 2020d).  Wash hands with soap and water for at least 20 seconds before and after interactions with other individuals (CDC, 2020b). |
| **18. Sessions in teaching rooms** | High | Ensure well-ventilated environment, with windows open fully where possible (CDC, 2020a; GHHIN, 2020; Morawska & Milton, 2020; Qian et al., 2020; UK Government, 2020b, WHO, 2020a).  Ensure that rooms are assessed to ensure that a minimum social distance of 2m can be maintained as much as possible or at least 1m if other mitigations measures are in place, such as wearing face coverings (UK Government, 2020d).  Disinfect surfaces between teaching sessions (CDC, 2020d; van Doremalen, et al., 2020).  Wear face coverings in accordance with current national and university policy and guidance (WHO, 2020b).  Wash hands with soap and water for at least 20 seconds before and after using shared room (CDC, 2020b; van Doremalen, et al., 2020). |
| **19. Working in a group** | High | Where possible, make use of virtual meeting platforms.  Adhere to rules about group sizes. Where possible minimize the number of university households involved in the group working (Nielsen & Sneppen, 2020; UK Government, 2020d).  Minimise contact time between individuals, keeping the use of shared spaces to less than 15 minutes when possible (ECDC, 2020).  If possible, undertake group working outside. If not possible, ensure well-ventilated environment (CDC, 2020a; GHHIN, 2020; Morawska & Milton, 2020; Qian et al., 2020; UK Government, 2020b, WHO, 2020a). Disinfect surfaces between sessions (CDC, 2020d; van Doremalen, et al., 2020).  Maintain a minimum social distance of 2m as much as possible or at least 1m if other mitigations measures are in place, such as wearing face coverings (UK Government, 2020d).  Wash hands with soap and water for at least 20 seconds before and after group work or contact with another individual (CDC, 2020b; van Doremalen, et al., 2020). |
| **20. Seating in teaching rooms** | High | Maintain a minimum social distance of 2m as much as possible or at least 1m if other mitigations measures are in place, such as wearing face coverings (UK Government, 2020d).  Wear face coverings in accordance with current national and university policy and guidance (WHO, 2020b).  Wash hands with soap and water for at least 20 seconds before and after group work or contact with another individual (CDC, 2020b; van Doremalen, et al., 2020).  Disinfect surfaces between teaching sessions (CDC, 2020d; van Doremalen, et al., 2020). |
| **21. Undertaking physical exams such as OSCEs/ consultation skills / physical manipulations** | High-low depending on use of PPE | Ensure 2m distance between students after exam  Examiner, student, and actor (if used) to keep 2m distance (UK Government, 2020d), or wear a mask and visor if social distancing cannot be maintained (WHO, 2020b).  Ensure well-ventilated environment, with windows open fully where possible (CDC, 2020a; GHHIN, 2020; Morawska & Milton, 2020; Qian et al., 2020; UK Government, 2020b, WHO, 2020a).  Students to wash hands before entering and when leaving each station (CDC, 2020b; van Doremalen, et al., 2020).  Examiner to wipe down any surfaces touched by students before next student enters the station (van Doremalen, et al., 2020).  Avoid mouth-to-mouth in emergency scenarios and instead provide single-use bag valve masks  Students to bring their own equipment (e.g. stethoscope and pen) to each station (van Doremalen, et al., 2020).  Use models/dummies instead of actors/patients for examinations where possible; sanitise models/dummies between users (CDC, 2020d; van Doremalen, et al., 2020). |
| **22. Provision of ad hoc in-person support (e.g. advisors, last minute support etc.)** | High | If possible and appropriate, provide support outside or using video conferencing. If not possible, ensure support is given in a well-ventilated environment (CDC, 2020a; GHHIN, 2020; Morawska & Milton, 2020; Qian et al., 2020; UK Government, 2020b, WHO, 2020a).  Adhere to rules about group sizes (Nielsen & Sneppen, 2020).  Minimise contact time between individuals, keeping the use of shared spaces to less than 15 minutes when possible (ECDC, 2020).  Maintain a minimum social distance of 2m as much as possible (UK Government, 2020d) or at least 1m if other mitigations measures are in place, such as wearing face coverings  Wash hands with soap and water for at least 20 seconds before and after in-person meetings (CDC, 2020b; van Doremalen, et al., 2020). |

**References**

Centers for Disease Control and Prevention. (2020a). Keeping the workplace safe, available at: <https://www.cdc.gov/coronavirus/2019-ncov/downloads/workplace-school-and-home-guidance.pdf>

Centers for Disease Control and Prevention. (2020b). Handwashing: Clean Hands Save Lives. When and How To Wash Your Hands, 24 November 2020, available at: <https://www.cdc.gov/handwashing/when-how-handwashing.html>

Centers for Disease Control and Prevention. (2020c). Cleaning and Disinfection for Households, 10 July 2020, available at: <https://www.cdc.gov/coronavirus/2019-ncov/prevent-getting-sick/cleaning-disinfection.html>

European Centre for Disease Prevention and Control. (2020). Contact tracing: Public health management of person, including healthcare workers, having had contact with COVID-19 cases in the European Union. ECDC, Stockholm, available at: <https://www.ecdc.europa.eu/sites/default/files/documents/covid-19-public-health-management-contact-novel-coronavirus-cases-EU.pdf>

Global Heat Health Information Network, (2020). Do air conditioning and ventilation systems increase the risk of virus transmission? If so, how can this be managed? Available at: <http://www.ghhin.org/heat-and-covid-19/ac-and-ventilation>

Macias, A. E., de la Torre, A., Moreni-Espinosa, S., Leal, P. E., Bourlon, M. T. & Ruiz-Palacios G. M. (2010) Controlling the novel A (H1N1) influenza virus: don’t touch your face! *The Journal of Hospital Infection, 73*(3), 280-281, doi.org/10.1016/j.jhin.2009.06.017.

Morawska, L. & D. K. Milton. (2020). It is time to address airborne transmission of Coronavirus Disease 2019 (COVID-19). *Clinical Infectious Diseases*, *71*(9), 2311-2313, doi.org/10.1093/cid/ciaa939

Nielsen, B. F., & Sneppen, K. (2020). Superspreaders provide essential clues for mitigation of COVID-19. *MedRxiv*, preprint doi.org/10/1101/2020/09/15/20195008

Qian, H., Miao, T., Liu, L., Zheng, X., Luo, D., & Li, Y. (2020). Indoor transmission of SARS-CoV-2. *MedRxiv,* pre-print doi.org/10.1101/2020.04.04.20053058

UK Government. (2020a). Celebrating religious festivals during coronavirus (COVID-19). *Ministry of Housing, Communities & Local Government*, UK Government. 10 December 2020, available at <https://www.gov.uk/government/publications/covid-19-guidance-for-the-safe-use-of-places-of-worship-during-the-pandemic-from-4-july/celebrating-religious-festivals-during-coronavirus-covid-19>

UK Government. (2020b). Coronavirus (COVID-19): safer travel guidance for passengers. *Department for Transport*, UK Government, 9 December 2020, available at <https://www.gov.uk/guidance/coronavirus-covid-19-safer-travel-guidance-for-passengers>

UK Government. (2020c). Stay at home: guidance for households with possible or confirmed coronavirus (COVID-19) infection. Coronavirus (COVID-19): safer travel guidance for passengers. *Public Health England*, UK Government, 5 November 2020, available at <https://www.gov.uk/government/publications/covid-19-stay-at-home-guidance/stay-at-home-guidance-for-households-with-possible-coronavirus-covid-19-infection>

UK Government. (2020d). Local restriction tiers: what you need to know. *Department of Health and Social Care*, UK Government, 30 November 2020, available at <https://www.gov.uk/guidance/local-restriction-tiers-what-you-need-to-know>

UK Government. (2020e). Keeping workers and clients safe during COVID-19 in close contact services. *HM* Government. 5 November 2020, available at <https://assets.publishing.service.gov.uk/media/5ef2889986650c12970e9b57/working-safely-during-covid-19-close-contact-041120.pdf>

van Doremalen, N., Bushmaker, T., Morris, D. H., Holbrook, M. G., Gamble, A., Williamson, B. N., Tamin, A., Harcourt, J. L., Thornburg, N. J., Gerber, S. I., Lloyd-Smith, J. O., de Wit, E., Munster, V. J. (2020). Aerosol and Surface Stability of SARS-CoV-2 as Compared with SARS-CoV-1. *New England Journal of Medicine*, *382*:1564-1567, doi.org/10.1056/NEJMc2004973

Wikipedia. (2020) Arm span. Available at: <https://en.wikipedia.org/wiki/Arm_span>

World Health Organisation. (2020a). Coronavirus disease (COVID-19): Ventilation and air conditioning in health facilities. *World Health Organisation,* 29 July 2020, available at <https://www.who.int/news-room/q-a-detail/coronavirus-disease-covid-19-ventilation-and-air-conditioning-in-health-facilities>

World Health Organisation. (2020b). Advice on the use of masks in the context of COVID-19. *World Health Organisation*, 5 June 2020, WHO reference number: WHO/2019-nCov/IPC_Masks/2020.4, available at https://apps.who.int/iris/rest/bitstreams/1279750/retrieve
